# Integrative oncology: a Canadian and international perspective

**DOI:** 10.3747/co.v15i0.292

**Published:** 2008-08

**Authors:** S.M. Sagar, A.M. Leis

We are pleased to present this special supplement of *Current Oncology,* which is generously financed by a grant from the Lotte and John Hecht Memorial Foundation. Integrative oncology is both a science and a philosophy that focuses on the complexity of the health of cancer patients and proposes a multitude of approaches to accompany the conventional therapies of surgery, chemotherapy, molecular therapeutics, and radiotherapy to facilitate health. As such, integrative oncology involves thinking outside the box, and so we are indeed fortunate to have attracted a plethora of manuscripts from innovative leaders both of Canadian and of international cancer treatment and control services.

In line with the modern approach to media communications, we have “integrated” this issue with manuscripts published on the *Current Oncology* Web site. Readers will also have the opportunity to view and listen to slide presentations submitted to the Integrating Wellness into Cancer Care Conference held at the University of Toronto, October 4–5, 2007. The conference was organized by Dr. Paul Fortin in memory of his wife Dr. Veronique Benk. Veronique was a radiation oncologist, clinician, and researcher who specialized in breast cancer, and she was devoted to her patients. Her personal experience of breast cancer and myeloid leukemia was transformative, and she embraced a wider approach to cancer treatment. That approach prioritized state-of-the-art medical care with a new emphasis on spirituality, wellness, and quality of life. The conference was sponsored by a non-restricted educational grant from the Lotte and John Hecht Memorial Foundation, CV Technologies, Astra/Zeneca Pharmaceuticals, Pfizer Pharmaceuticals, Novartis Pharmaceuticals, Wellspring, and the University of Toronto Department of Radiation Oncology, with special thanks to Drs. Mary Gospodarowicz and Pamela Catton.

## PURSUING THE INTEGRATIVE PATH

So, what is cam?

Complementary and alternative medicine (cam) is an umbrella term encompassing a group of diverse medical and health care systems, practices, and products that are not always considered part of conventional medicine 1. Yet in traditional healing systems, the power of the mind and other non-pharmaceutical interventions are integral to the treatment of ill health.

Promotion, maintenance, monitoring, or restoration of health is the goal of cam use. According to the U.S. National Cancer Institute, cam includes whole medical systems (for example, Traditional Chinese Medicine), mind–body medicine (meditation, for instance), biologically-based practices (natural health products, among others), manipulative and body-based therapies (for example, massage), and energy therapies (*qi gong,* for instance) 1.

An approach is called “complementary” when it is used adjunctively to conventional treatments, with the intent to enhance the body’s natural abilities to heal. Modalities used instead of conventional treatments are labelled “alternative” therapies. The term “alternative” means that treatments outside of conventional medicine are used to treat the disease. Depending on intent, some therapies can be considered either complementary or alternative. It is therefore important always to clarify the intent of the patient considering cam use. Studies have consistently found that most cancer patients use cam as a complement to their conventional treatment 2,3.

The lack of consensus around terminology and definitions makes it difficult to accurately assess the prevalence of cam use. For example, the list of products and therapies designated as cam continually changes as therapies that are proved to be safe and effective are integrated into conventional health care and as new, untested ones emerge 4.

An increasing number of people living with cancer are using therapies in addition to those prescribed by conventional health care providers 5,6. In the literature, an explosion of cam surveys has suggested that utilization rates by cancer patients fall between 40% and 60% 7. For example, with the inclusion of prayer in the cam list, the prevalence of cam use in the United States is estimated at 62%; minus prayer, it is about 36% 8.

Integrative oncology does not usually incorporate prayer into its definition of therapies, preferring to classify prayer in a religious domain, but the concept of prayer as a mind–body intervention illustrates the challenge of defining a cam therapy 9. Throughout the cancer trajectory, higher use of cam is consistently found during chemotherapy to mitigate adverse effects, after conventional cancer treatment to boost energy, during survivorship to foster wellness, and during the last months of life to control symptoms 10.

For this special issue, the term “cam” is being used because of its worldwide recognizability as a label linked to traditional medicine. Given the specific focus of this issue on integrative medicine, cam uniquely refers to the complementary side—in other words, to something used in conjunction with conventional oncology treatments. The powerful synergy of the holistic approach of complementary medicine together with biomedical cancer treatments is central to the purpose of integrative oncology. The combination permits dysfunctional physical, mental, emotional, and spiritual symptoms to be treated and thereby fully addresses the healing needs of cancer patients in a tailored fashion.

## IN THIS SUPPLEMENT

In his article, Dr. Simon Sutcliffe discusses the importance of combining science and evidence-based medicine with individual and societal values and of integrating values into a process of holistic care. To quote Sutcliffe, the goal is ultimately “to achieve a responsive, efficient, effective, and sustainable system to improve health and control cancer (as a process, not as an event).” He expands his argument that complex problems require a more sophisticated approach than can be achieved through scientific reductionism, and that the multifaceted perspectives of the patient must be part of the decision-making process in designing health policy.

Drs. Jacqueline Bender and Alejandro Jadad extend the perspective of patients’ values and education through individuals socializing over the Internet. They discuss the potential for empowerment and how that empowerment may modify the relationships of patients with their health care providers. Dr. Alastair Cunningham emphasizes the existential crisis that patients endure when they receive a diagnosis of cancer and speaks of the importance of psychological healing as part of the process of restoring health. And Dr. Mary Vachon expands on the notion of spirituality and meaning for cancer patients in her article “The Soul’s Wisdom: Stories of Living and Dying” (e-manuscript on the Web).

How is outcome to be evaluated in an integrative oncology program? Dr. Stephen Sagar discusses various health outcome domains and patient satisfaction, and points to some validated measurement tools. For a detailed source of measurement tools, readers are also directed to the new online IN-CAM Outcomes Database (www.outcomesdatabase.org) organized by Dr. Marja Verhoef.

Dr. Sagar and Dr. Raimond Wong together provide an educational article on integrative oncology research and regulation (e-manuscript on the Web). Further e-manuscripts recount the experiences of two international integrative oncology programs: Dr. Jane Maher, Chief Medical Officer of MacMillan Cancer Support, describes the Lynda Jackson Macmillan Centre at Mount Vernon Hospital in Northwood, United Kingdom, and Dr. Gary Deng describes the Integrative Medicine Service at Memorial Sloan–Kettering Hospital, headed by Dr. Barrie Cassileth, in New York City, United States. Both centres have pioneered similar models in other countries.

Attendees of the University of Toronto Integrating Wellness into Cancer Care Conference participated in a workshop titled “How to Put Wellness on the Prescription Pad”, and in another e-manuscript, Dr. Fortin presents a summary of the discussions that took place.

A group of manuscripts from the Cancer, Complementary and Alternative Medicine (ccam) team, a multidisciplinary group of Canadian scientists, presents some of their work in evaluating the role of complementary therapies for cancer care.

Dr. Ann Leis discusses the scope of integrative oncology and the need for evidence to support the integration of complementary therapies into cancer care. She concludes that “a whole-systems framework to the development of the evidence base for integrative oncology can guide the development of evidence that respects the complex nature of many complementary and integrative practices and their underlying principles of care delivery.”

In her manuscript “Talking to Cancer Patients About Complementary Therapies,” Marja Verhoef concludes that discussing cam with patients is the physician’s responsibility and that it will facilitate evidence-based, patient-centred cancer care.

Dr. Lynda Balneaves uses research from the Canadian health literature to address the issue of patient decision-making. In her discussion, she says that “decision to use, or not to use, cam is not a one-time whimsical decision; instead, it is a decision that leads cancer patients to reflect on their unique personal and social context and to ponder how cam may fit with their values, beliefs, and specific health care needs. As the individual and social contexts of patients change, the appropriateness of select cam therapies in their treatment regimen also changes. Decisions about cam are not static; rather, they are dynamic entities that require assessment and follow-up by health professionals throughout a patient’s illness.”

Why do some patients decline conventional evidence-based therapies and pursue alternative non-proven options? Dr. Verhoef concludes that “poor doctor–patient communication, the emotional impact of the cancer diagnosis, perceived severity of conventional treatment side effects, a high need for decision-making control, and strong beliefs in holistic healing appear to affect the decision by patients to decline some or all conventional cancer treatments.” The “tendency by doctors to dichotomize patient decisions as rational or irrational may interfere with the ability of the doctors to respond with sensitivity and understanding,” she continues.

Doctor of naturopathic medicine Dugald Seeley, together with Dr. Doreen Oneschuk, discusses the important topic of interactions of natural health products with biomedical cancer treatments.

These subjects require knowledgeable physicians and a modification of medical school curricula, a task that is being undertaken by the CAM in UME Project (www.caminume.ca/about.html) and the Consortium of Academic Health Centers for Integrative Medicine (www.imconsortium.org).

What, then, are the current integrative practices of Canadian health care professionals? Dr. Alison Brazier uses an interpretive–description research design, with a series of in-depth qualitative interviews, to highlight two main strategies: acting as an integrative cancer guide and collaborating with other health care professionals.

Mind–body techniques derived from Eastern mysticism have become an important part of some integrative oncology programs. In a research paper that evaluates an Iyengar yoga program, Ms. Meghan Duncan finds an overall improvement in the well-being of cancer patients (e-manuscript on the Web). This research contributes further to the evidence that some techniques derived from Eastern spirituality can help some patients cope with cancer and its treatment.

## THE WAY FORWARD

The future of complementary therapies lies in mainstream medicine, but only if those therapies are based on scientific understanding and evidence of effectiveness. A willingness to discard therapies that fail to be proved effective in clinical studies is vital. Accepted therapies must also be seen to be safe and cost-effective.

A comprehensive cancer program should integrate surgery, chemotherapy, radiotherapy, and targeted molecular therapies with meaningful psycho-spiritual, psychological, and physical supportive therapies, and an investigative program of botanicals. New technology is facilitating the quality-controlled preparation of simple and complex mixtures of phytochemicals that are being investigated as biologic response modifiers. International collaboration between North American, European, and Asian universities and interested pharmaceutical companies is encouraging the development of multi-targeted therapies using traditional herbs. During a recent visit by S.M.S. to Fudan University in Shanghai, China, that institution ratified a collaborative research agreement with the M.D. Anderson Hospital (the largest international cancer centre) and signed a new collaborative agreement with the Institut Gustav Roussy (Europe’s largest cancer centre), under the umbrella of integrative oncology. Video extracts of the Shanghai conference of the Society for Integrative Oncology can be found on the *Current Oncology* Web site. Further information on the Society for Integrative Oncology and its conferences (including abstracts from its recent conference held in Shanghai) can be found at www.integrativeonc.org.

## YOUR PART IN THE DISCUSSION

Readers are invited to evaluate this special supplement by answering a short survey on the *Current Oncology* Web site. The editors appreciate your interest in this supplement and will similarly appreciate receiving your comments. We would like to publish those comments on the Web site; the option of anonymity for specific comments is available.
